# Biodegradation Mechanisms and Sustainable Governance of Marine Polypropylene Microplastics

**DOI:** 10.3390/nano16030163

**Published:** 2026-01-26

**Authors:** Haoze Lu, Dongjun Li, Lin Wang

**Affiliations:** 1 Miami College, Jinming Campus, Henan University, Kaifeng 475004, China; luhaoze@henu.edu.cn; 2 Zhoukou Hydrology and Water Resources Forecasting Sub-Center, Zhoukou 466001, China; lidongjun@126.com; 3 College of Geographical Sciences, Faculty of Geographical Science and Engineering, Henan University, Zhengzhou 450046, China

**Keywords:** polypropylene microplastics, biodegradation mechanism, microbial community, environmental remediation, marine pollution

## Abstract

Polypropylene microplastics (PP-MPs) represent a persistent class of marine pollutants due to their hydrophobicity, high crystallinity, and resistance to environmental degradation. This review summarizes recent advances in understanding the environmental behavior, physicochemical aging, and ecotoxicological risks of PP-MPs, with emphasis on microbial degradation pathways involving bacteria, fungi, algae, and filter-feeding invertebrates. The biodegradation of PP-MPs is jointly regulated by environmental conditions, polymer properties, and the structure and function of plastisphere communities. Although photo-oxidation and mechanical abrasion enhance microbial colonization by increasing surface roughness and introducing oxygenated functional groups, overall degradation rates remain low in marine environments. Emerging mitigation strategies include biodegradable polymer alternatives, multifunctional catalytic and adsorptive materials, engineered microbial consortia, and integrated photo–biodegradation systems. Key research priorities include elucidating molecular degradation mechanisms, designing programmable degradable materials, and establishing AI-based monitoring frameworks. This review provides a concise foundation for developing ecologically safe and scalable approaches to PP-MP reduction and sustainable marine pollution management.

## 1. Introduction

Plastic pollution has emerged as one of the most pressing global environmental issues. It is estimated that over 10 million tons of plastic waste enter marine systems annually, where prolonged exposure to sunlight, oxidation, and mechanical abrasion gradually fragment macroplastics into smaller particles. Transported by ocean currents and wind, plastic debris disperses globally and continues to break down over time, resulting in its widespread presence across various size fractions. While meso- and macroplastics consist of particles larger than 5 mm in diameter, microplastics—defined as particles smaller than 5 mm—represent a pervasive and environmentally consequential outcome of this continuous degradation process [1]. Research has found that in the marine environment, polyethylene accounts for 23% of plastic debris, followed by polyamide (20%), polypropylene (13%), and polystyrene (4%) [2]. These MPs are widely distributed in seawater and sediments, accounting for approximately 92.4% of plastic particles in the ocean [3,4]. Studies have demonstrated that microplastics not only impose physical stress such as blockage or abrasion on marine organisms but also adsorb various persistent organic pollutants, posing risks to ecosystem stability and human health [5].

Polypropylene (PP) is one of the most extensively used polymers worldwide, accounting for about 16% of global plastic consumption [6]. Its low density, high crystallinity, and hydrophobic surface confer strong environmental persistence and resistance to degradation. In marine systems, PP microplastics (PP-MPs) predominantly occur in the upper 5–10 m of the water column and can be transported over long distances by currents, winds, and tidal forces [7]. Through biofilm formation, these particles gradually sink and accumulate in seabed sediments. Such vertical and horizontal redistribution enables PP MPs to persist across multiple ecological compartments, similar to other persistent microplastics, which collectively complicates marine pollution management.

Beyond their physical impacts, PP-MPs exert notable ecotoxicological effects. Experimental evidence indicates that UV-weathered PP leachates significantly inhibit the growth of marine algae, while benthic organisms that ingest PP-MPs may transfer them through the food web to higher trophic levels, potentially causing gastrointestinal obstruction, disrupted energy metabolism, and altered immune responses [7,8]. In addition, the hydrophobic surfaces of PP-MPs facilitate the adsorption of organic contaminants such as polychlorinated biphenyls and pesticide residues [7]. Under certain environmental conditions, these pollutants may be subsequently released, increasing their bioavailability and enhancing toxic accumulation [9]. Consequently, PP-MPs function simultaneously as physical pollutants and as vectors for chemical contaminants in marine environments.

In summary, the complex distribution behavior, environmental persistence, and combined pollution effects of PP-MPs pose significant challenges to current marine pollution control efforts. This review aims to systematically examine the physicochemical properties, ecotoxicological mechanisms, and biodegradation pathways of PP-MPs in marine environments. Furthermore, it explores sustainable mitigation strategies to provide a scientific foundation for promoting the long-term health and sustainable development of marine ecosystems.

## 2. Biodegradation Mechanisms of Polypropylene Microplastics

**Figure 1:**
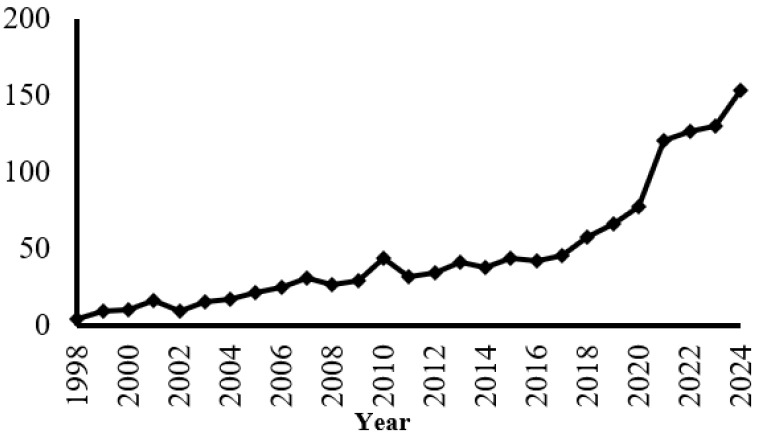
Number of publications related to PP biodegradation since 1998 [3].

With the expanding distribution of PP-MPs in marine environments, their recalcitrance and environmental persistence have become major global ecological concerns. As an environmentally friendly remediation strategy, biodegradation has gained increasing attention in recent years. A bibliometric analysis based on the Web of Science database using the keywords “polypropylene” and “biodegradation” shows a continuous growth in related publications from 1998 to 2024, with a marked acceleration in the past five years, indicating the rapid development of this research field (Figure 1).

**Figure 2:**
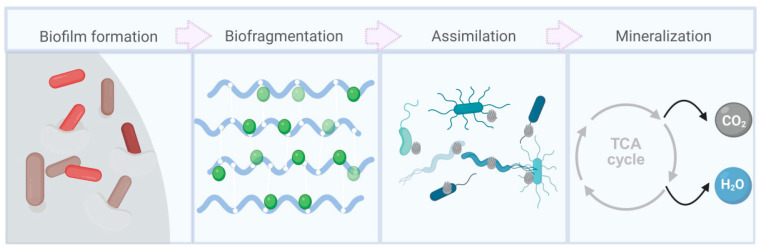
The microbial degradation pathways of PP-MPs [3].

PP biodegradation is mainly driven by the metabolic functions of bacteria, fungi, and algae, involving biofilm development, enzymatic breakdown, and mineralization (Figure 2). These processes gradually convert high-molecular-weight polymers into oligomers, small molecules, and eventually carbon dioxide and water.

### 2.1. Bacteria-Mediated Degradation

**Figure 3:**
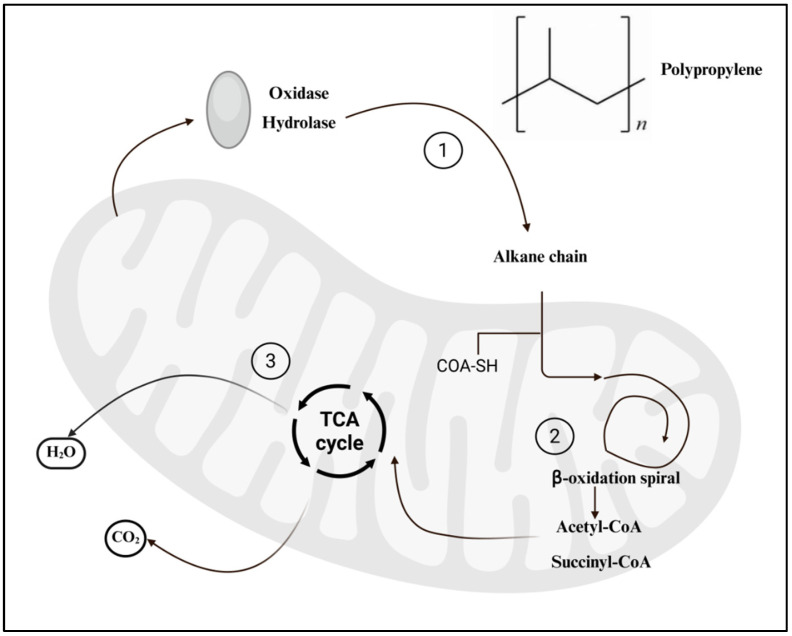
Major processes in bacteria-mediated PP-MPs degradation [13]. CoA: Coenzyme A; -SH: Sulfhydryl group; and TCA: Tricarboxylic acid cycle.

In marine ecosystems, bacteria are considered the most metabolically active and potentially effective microbial group involved in PP microplastic degradation (Figure 3). The degradation process generally includes four stages: biodeterioration, biofragmentation, assimilation, and mineralization [10].

During the initial biodeterioration phase, bacterial enzymes including lipases, ureases, and proteases attack the PP surface, promoting oxidation and chain scission that generate oxygenated functional groups and destabilize the polymer structure. In the subsequent biofragmentation stage, hydrolytic and oxidoreductive enzymes further break the polymer chains into oligomers and monomers. The formation of free radicals is a key step that facilitates carbon-chain cleavage and structural destabilization. During assimilation and mineralization, these low-molecular-weight intermediates serve as carbon sources for microbial metabolism, ultimately being converted into cellular biomass, CO_2_, and CH_4_ [11]. However, PP’s hydrophobicity limits the diffusion of degradation intermediates across microbial membranes, making the process highly dependent on synergistic interactions among bacterial communities.

Photochemical weathering significantly enhances bacterial colonization and degradation efficiency. Davidov et al. [12] demonstrated that PP exposed to xenon or UV-B irradiation exhibits reduced hydrophobicity and increased surface polarity, promoting biofilm formation and providing more reactive sites for microbial attack. Previous studies have also suggested that PP degradation follows a mechanism similar to that of polyethylene (PE) [3]. Although no highly efficient or polypropylene-specific enzyme has been isolated to date, insights from polyethylene mineralization suggest that polypropylene biodegradation likely proceeds through a similar mechanism involving oxidation, chain scission, and ultimate microbial utilization [3].

Bacterial communities isolated from different marine regions show substantial variation in degradation performance. Table 1 lists dominant taxa detected on PP microplastic biofilms at representative sampling sites in marine environments. Future research should focus on isolating and characterizing key enzymes, elucidating structural homologies with polyethylene- and polystyrene-degrading enzymes, and genetically engineering microbial strains to enhance biodegradation efficiency.

### 2.2. Fungal Degradation

Fungi play an important role in plastic biodegradation due to their secretion of diverse oxidoreductases and hydrolases. They are widely distributed in soils, marine habitats, and detritus-rich environments and are capable of decomposing complex natural polymers such as lignin and cellulose [26]. However, the β-methyl branching in PP chains enhances its chemical stability, making fungal degradation relatively difficult.

**Figure 4:**
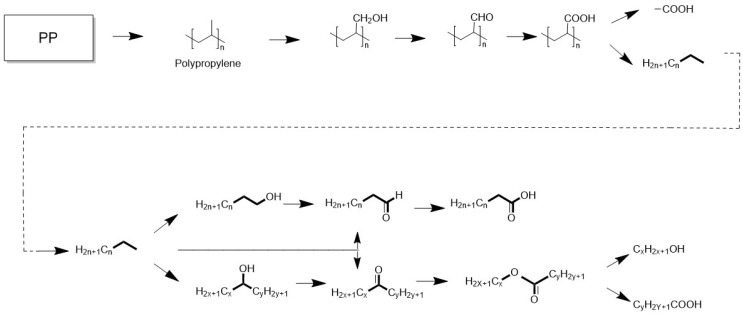
Metabolic pathway of PP degradation by strain FB1 [27].

Despite this, several studies have demonstrated partial fungal degradation of PP under specific conditions. Su et al. [27] identified a marine fungus, Alternaria sp. FB1, capable of degrading untreated PP. Gene knockout and transcriptomic analyses revealed its putative metabolic pathways, which include three major steps: colonization, depolymerization, and metabolic utilization (Figure 4).

During colonization, fungi secrete hydrophobic proteins that enhance substrate adhesion. In the depolymerization stage, laccases and peroxidases disrupt PP’s hydrophobic backbone and generate reactive oxygen species, facilitating side-chain cleavage. Subsequently, dehydrogenases and alcohol dehydrogenases drive terminal and subterminal oxidation, converting polymer fragments into metabolically accessible intermediates such as fatty acids, esters, and alcohols.

### 2.3. Algal and Invertebrate Involvement

Compared with bacteria and fungi, the role of algae in PP microplastic degradation remains understudied. Algae possess advantages such as independence from external organic carbon sources and adaptability to diverse marine ecological niches [13]. Existing studies suggest that algal enzymes, such as lignin-degrading oxidases and extracellular polysaccharidases, can promote surface cracking of plastics and participate in physico-chemical degradation during biofilm formation [3].

Viel et al. [28] reported strong interactions between microplastics and Spirulina, and Stabili et al. [29] proposed a macroalgae-based bioremediation strategy for in situ microplastic capture. Although PE TaseR280A-FLAG, a PET-degrading algal enzyme, is currently the only experimentally validated marine algal depolymerase [30], its discovery provides a valuable reference for exploring algal degradation of PP.

**Figure 5:**
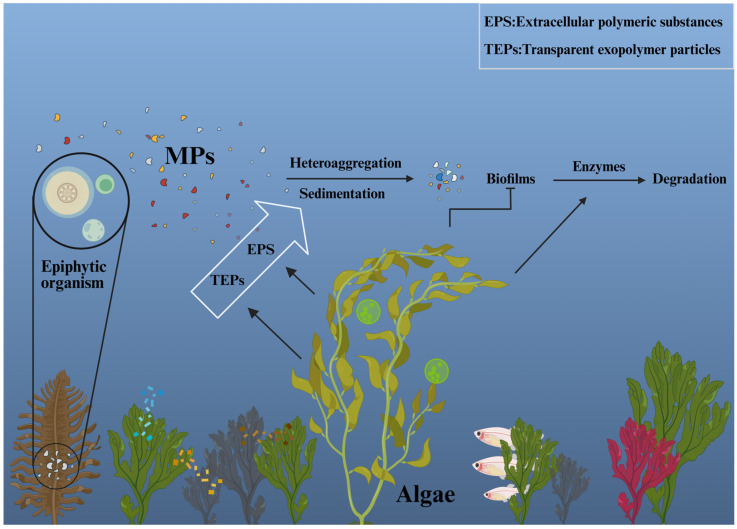
Mechanisms of microplastic capture, transport, and degradation mediated by marine algae. EPS: extracellular polymeric substances; TEPs: EPS-derived transparent exopolymer particles.

Marine macroalgae capture MPs through physical processes such as adhesion, embedding, encapsulation, and entanglement, primarily facilitated by polysaccharide-rich mucilage layers and epibiotic matrices, with coexisting microalgae further enhancing MP accumulation (Figure 5). Following capture, extracellular polymeric substances (EPS) and EPS-derived transparent exopolymer particles (TEPs) promote heteroaggregation and sedimentation of MPs, thereby regulating their transport and environmental fate. Meanwhile, algal-associated biofilms colonize MP surfaces and secrete degrading enzymes (e.g., PETase and lipases), altering MP surface properties (e.g., increased hydrophilicity and surface cracking) and driving a sequential “colonization–fragmentation–assimilation” biodegradation pathway. These processes are jointly regulated by MP properties, algal species specificity, EPS production, and environmental conditions, while dense algal coverage may reduce photodegradation of MPs [31].

Recent evidence shows that filter-feeding invertebrates, including bivalves and zooplankton, may aid in microplastic biotransformation via ingestion, supporting their partial elimination from marine systems [32]. This suggests that the biological transformation of microplastics is not limited to microbial degradation, but also involves other organisms, such as filter-feeding invertebrates, which contribute to the breakdown process. Overall, the degradation of PP microplastics depends heavily on biofilm formation and oxidative cleavage, processes that are significantly influenced by microbial and other biological interactions. The synergistic effects between microorganisms and filter-feeding invertebrates, for example, may enhance the overall efficiency of microplastic degradation.

However, microplastic ingestion by bivalves can impair population viability through sublethal effects on fitness. Exposure reduces nutrient assimilation, diverting energy from reproduction and compromising gametogenesis and larval development. Concurrently, MPs alter key physiological and behavioral functions, which cumulatively increase mortality risk and threaten long-term population stability [33]. Cole et al. [34] demonstrated that zooplankton are capable of ingesting some specific sizes of marine microplastic debris in the absence of natural food. This ingestion can lead to impaired individual health and physiological function, facilitate the trophic transfer of contaminants to higher predators, and result in the egestion of fecal pellets containing microplastics, and the negative impact may dominate in the current environment. The filter-feeding invertebrates, including bivalves and zooplankton, readily ingest microplastics. However, due to the recalcitrance of PP, significant biotransformation is limited. Instead, these organisms primarily act as biological pumps; by packaging ingested MPs into dense fecal pellets, they accelerate the vertical transport of MPs from the water column to benthic sediments, thereby altering their environmental distribution rather than degrading them [32].

## 3. Key Factors Influencing the Biodegradation of Polypropylene

### 3.1. Environmental Conditions

Marine environmental conditions—characterized by low temperatures, limited light penetration, reduced oxygen availability, and high salinity—typically hinder PP degradation. Temperature is a fundamental regulator of microbial metabolism and enzymatic activity. A decrease in temperature markedly suppresses catalytic efficiency and biofilm formation [13]. Light, particularly ultraviolet (UV) radiation, is another crucial driver. UV exposure generates free radicals and surface oxidation, accelerating polymer weathering and increasing microbial attachment sites, thereby indirectly promoting biodegradation.

Oxygen concentration plays a decisive role in sustaining aerobic microbial activity. Adequate oxygen supply facilitates initial oxidation and subsequent mineralization, whereas anaerobic conditions significantly reduce degradation efficiency. In addition, nutrient availability—especially carbon and nitrogen sources—directly affects microbial growth and enzyme secretion, thereby modulating degradation rates. Chemical parameters such as dissolved oxygen, salinity, pH, and dissolved organic carbon content may alter polymer surface charge and influence microbial adhesion.

PP degradation in the marine environment varies significantly with depth, primarily due to differential exposure to ultraviolet (UV) radiation, oxygen availability, and physical weathering processes. Buoyant PP tends to accumulate at the surface, where intense UV radiation initiates photochemical degradation, leading to polymer embrittlement, cracking, and fragmentation [9]. This process is somewhat moderated in the water column, as dissolved salts, organic compounds, and suspended particles scatter and absorb UV light, reducing its penetration [35]. In deeper waters and sediments, where UV exposure is minimal, degradation proceeds through slower pathways, predominantly microbial activity and physical abrasion, resulting in extended environmental persistence. Wave action is a key driver of mechanical weathering. For PP, while wave-induced shear, turbulence, and abrasion alone cause limited degradation, they act synergistically with UV radiation: UV initially weakens the polymer matrix, and subsequent wave stresses efficiently propagate cracks and accelerate fragmentation [35]. This photo-mechanical synergy significantly promotes particle size reduction, facilitating the breakdown of PP from macro- to micro- and nanoplastics.

Koike et al. [24] reported that PP MPs degrade faster in midwater layers than in surface waters, suggesting that relatively stable temperature and dissolved oxygen conditions at depth may favor bacterial colonization and metabolism. Notably, extreme weather events, such as storms and hurricanes, induce vigorous vertical mixing and sediment resuspension. This leads to increased mechanical abrasion via collisions with suspended particles, which can physically scour established biofilms more effectively than regular tidal flows, potentially interrupting the biodegradation process [36].

### 3.2. Material Properties

The inherent physicochemical features of PP-MPs largely determine their susceptibility to biodegradation. Their diverse morphologies, including fragments, pellets, films, and fibers, differ in specific surface area and crystallinity, thereby shaping microbial colonization and enzymatic interaction. In general, smaller particles with larger specific surface areas exhibit faster degradation rates [37]. In addition, polymer molecular weight, type of functional groups, and surface energy affect enzymatic hydrolysis and oxidation reactions [38].

Smith et al. [39], using wastewater treatment plant samples, found that the presence of PP-MPs did not significantly influence cellulose biodegradation, suggesting that non-degradable polymers may function merely as inert substrates. This finding highlights that PP environmental degradation is primarily determined by its intrinsic structural properties and degree of weathering rather than by competition with other organic substrates. As aging progresses, surface polarity increases and roughness intensifies, creating more favorable conditions for microbial adhesion and biofilm formation. Furthermore, PP is a carbon-rich but nutrient-poor substrate, lacking essential elements like nitrogen and phosphorus. This stoichiometric mismatch forces colonizing microbes to scavenge nutrients from the surrounding water, making nutrient availability a critical bottleneck that diminishes the substrate’s attractiveness and limits biodegradation rates.

### 3.3. Microbial Communities and Ecological Interactions

Microbial colonization and community succession constitute the primary biological drivers of PP-MP biodegradation. Microplastic surfaces provide a stable substrate for microbial attachment, giving rise to a specialized ecological habitat termed the “plastisphere” [29]. Degradation efficiency is closely linked to microbial diversity, functional complementarity, and ecological interactions. Initial colonization is influenced by polymer type, degree of weathering, and environmental parameters. Davidov et al. [12] observed that *Alcanivorax* exhibited preferential colonization on polyolefin surfaces such as PE and PP, suggesting a potentially significant role in PP degradation.

Ecological relationships among microbes—competition, predation, and symbiosis—shape community structure and influence degradation capacity. Karkanorachaki et al. [40] noted that although degradation rates fluctuate over time, plastisphere communities tend to maintain low diversity yet stable composition, reflecting a dynamic but balanced state [27]. Wang et al. [41] further demonstrated that when microplastic-associated bacteria were transferred from aquaculture waters to seagrass meadows, exogenous biodegradation occurred, with PP-MPs showing the most pronounced changes. This highlights the significant influence of environmental migration and community exchange on PP degradation pathways.

Overall, the biodegradation of PP microplastics is a complex process shaped by the coupling of environmental factors, material properties, and microbial ecological interactions. Temperature and light regulate reaction kinetics, polymer structure dictates bioavailability, and microbial community succession controls enzymatic activity and degradation potential. Future research should focus on multi-factor coupling mechanisms and utilize multi-omics approaches (metagenomics, metabolomics) to elucidate the functional networks of plastisphere communities.

## 4. Prospects for Application and Governance Strategies

### 4.1. Research Methods and Analytical Techniques

Current research on the biodegradation of PP-MPs primarily involves three methodological components: microbial screening, molecular identification, and material characterization. Typically, soil and water samples are collected from coastal regions heavily affected by plastic pollution. These samples are enriched using selective media containing PP MPs as the sole carbon source, followed by isolation of pure strains through serial dilution and plate spreading [23]. Highly efficient degraders are subsequently subjected to 16S rRNA sequencing and phylogenetic analysis to determine their taxonomic identity and potential functional traits.

Following biodegradation experiments, multiple characterization techniques are employed to evaluate structural modifications in PP. ATR-FTIR spectroscopy is widely used to monitor changes in surface functional groups, while micro-Fourier transform infrared spectroscopy (μ-FTIR) provides spatially resolved chemical mapping of microplastic surfaces, enabling the detection of localized oxidative or hydrolytic degradation products. Scanning electron microscopy (SEM) provides high-resolution images of surface morphology and biofilm architecture; X-ray photoelectron spectroscopy (XPS) further elucidates elemental composition and chemical bonding states within the top 10 nm of the polymer surface [42]. To gain deeper insights into the degradation mechanism, pyrolysis-gas chromatography-mass spectrometry (Py-GC-MS) is employed to analyze polymer breakdown products and quantify changes in molecular composition resulting from chain scission. Furthermore, whole-genome sequencing may be applied to identify relevant enzyme families and degradation-associated gene clusters.

In microplastic monitoring, infrared and Raman spectroscopy remain the dominant tools for identification and quantification due to their high spatial resolution. Recently, machine learning and deep-learning–based recognition systems have been developed for automated plastic detection through remote sensing, offering new opportunities for large-scale and real-time environmental monitoring [2].

### 4.2. Sustainable Governance Pathways and Emerging Technologies

#### 4.2.1. Application of Biodegradable Materials and Risk Assessment

As plastic pollution intensifies, promoting the widespread application of biodegradable materials has become a key strategy in global efforts to mitigate microplastic pollution. Lu et al. [43] reported that polypropylene-based fishing gear and aquaculture facilities are major contributors to marine plastic waste in Hong Kong and the South China Sea. In response, the European Union has mandated the preferential use of biodegradable materials in fisheries [44]. However, significant trade-offs remain among degradation performance, economic feasibility, and ecological safety. For example, Manfra et al. [45] demonstrated that polylactic acid (PLA), a biodegradable plastic, and PP microplastics exhibit comparable ecotoxicological impacts, highlighting that biodegradability alone does not ensure ecological safety. While biodegradable materials hold great potential for addressing marine plastic pollution, challenges remain regarding the compatibility of existing production infrastructure with their manufacturing processes. Currently, many plastic production lines are designed specifically for the production of conventional plastics such as polypropylene and polyethylene, relying on high-temperature and high-pressure polymerization processes. These facilities are optimized for materials with strong chemical stability and non-biodegradable properties, making them ill-suited for the production of biodegradable plastics. As a result, significant adjustments to existing production infrastructure may be necessary, including modifications to raw material formulations, process parameters, and equipment to meet the specific demands of biodegradable plastic production. But it is crucial to recognize that ‘biodegradability’ is highly context-dependent. Many commercial biodegradable plastics (e.g., PLA) require industrial composting conditions with high temperatures (>50 °C) to initiate hydrolysis, conditions that are virtually absent in the marine environment. Even for marine-degradable candidates like polyhydroxyalkanoates (PHAs), practical degradation rates in the open ocean are significantly slower than in laboratory settings due to low water temperatures and dilute microbial concentrations. Therefore, current biodegradable alternatives should not be viewed as a complete solution to marine pollution, and future material design must specifically target degradation triggers compatible with cold, saline marine conditions.

To ensure the successful manufacturing of biodegradable materials, infrastructure upgrades may involve several aspects. First, optimizing production processes is critical, particularly in adjusting temperature, pressure, and catalyst usage. The production of biodegradable plastics typically requires lower reaction temperatures and specific catalytic conditions, which differ from those used in conventional plastic production. Additionally, certain production stages may need to be redesigned to accommodate the different types of waste generated during the process, which may place new demands on existing waste management systems. Furthermore, upgrading or replacing equipment is necessary, especially in molding, cutting, and processing stages, to support the unique characteristics of biodegradable materials and ensure efficient and stable production processes.

Moreover, although biodegradable plastics theoretically offer a solution to plastic pollution, their production costs and environmental impact must be comprehensively evaluated. The production of biodegradable materials typically comes with higher costs, including raw material costs and the expenses associated with technological upgrades. Therefore, balancing the environmental benefits of biodegradable materials with their economic feasibility is a crucial factor for their large-scale adoption. In this context, integrating Life Cycle Assessment (LCA) and Ecological Risk Assessment (ERA) frameworks is particularly important. Crucially, these scientific insights must be translated into robust regulatory policies. Strengthening international legislation on plastic production standards, waste management protocols, and extended producer responsibility is paramount to effectively enforcing pollution reduction targets [46].

#### 4.2.2. Potential of Novel Adsorptive and Catalytic Materials and Ecological Remediation Using Microorganisms

The development of advanced adsorptive and catalytic materials, along with microbial-based ecological remediation, provides interdisciplinary solutions for addressing microplastic pollution, particularly in marine environments. Dutta et al. [47] designed a Cu-POM nanocluster-embedded interpenetrating polymer network that maintains high removal efficiency after five regeneration cycles, demonstrating excellent reusability. This material is capable of simultaneously adsorbing PP-MPs and removing heavy metal ions.

Meanwhile, microbial-based ecological remediation remains a core strategy for mitigating PP-MPs in marine ecosystems. Organic-rich habitats, such as mangroves, offer ideal environments for complex microbial consortia [48]. While individual microbial strains typically achieve limited PP degradation (The mass loss is approximately 4–6%) [22], mixed microbial consortia have shown a significant increase in degradation efficiency, achieving gravimetric weight losses of up to 13.1%, due to metabolic complementarity and enzymatic synergy [48]. It is noted that the degradation efficiency here is defined as the percentage of mass loss [(m0 − mt)/m0 × 100%]. By integrating innovative materials with biological remediation strategies, a more comprehensive approach can be developed for effectively controlling microplastic pollution.

#### 4.2.3. Integrated Degradation Mechanisms and Innovation Pathways

The degradation of PP microplastics is often governed by coupled processes involving photo-oxidation, thermal aging, and biological activity. Shariff et al. [49] demonstrated that PP/PLA blends can achieve significantly accelerated biodegradation while maintaining optimal mechanical properties at a blend ratio of 9:1. In addition, UV-induced weathering enhances biofilm formation and polymer fragmentation, establishing a cyclic “photo–bio–photoreactivation” degradation pathway [36].

Future governance strategies should emphasize multi-mechanism synergy and interdisciplinary integration, including: (1) development of “programmable degradation” materials combining advances in materials science and microbiology, (2) use of artificial intelligence to simulate environmental drivers and predict degradation efficiency, and (3) establishment of full-chain verification systems encompassing laboratory research, pilot-scale testing, and in situ marine validation. These efforts will accelerate the transition from passive treatment to intelligent, environmental-responsive degradation systems.

### 4.3. Future Research Directions and Policy Implications

Future research on PP-MP mitigation should center on material innovation, biodegradation mechanisms, and intelligent monitoring. At the material level, new low-risk polymers should be developed to reduce reliance on traditional chemical additives, thereby minimizing environmental footprints across production and use phases [50]. In the realm of biodegradation, techniques such as gene editing, protein engineering, and directed evolution can be employed to discover and optimize highly efficient degraders and key catabolic enzymes, enhancing PP degradability at the molecular level [3]. Additionally, next-generation monitoring will rely on cross-scale technologies that integrate remote sensing, infrared spectroscopy, and artificial intelligence, enabling real-time identification and source tracing of microplastics across spatial scales.

Policy and regulatory frameworks are essential for translating scientific innovation into practical governance. First, comprehensive evaluation and regulatory systems for biodegradable materials are needed to prevent “greenwashing”. Governments may promote innovation and industrialization through financial incentives and supportive policies, facilitating sustainable economic development [51]. Early removal of marine plastic waste, particularly fragile, high-risk polyolefins, should be prioritized to reduce microplastic generation. Converting recovered marine plastics into high-value materials can mitigate environmental impacts while supporting employment and coastal economies [52].

Importantly, policy implementation must be grounded in robust scientific evidence. For instance, although pro-oxidant additives such as P-Life can accelerate oxidative chain scission in polyolefins, they do not enhance marine biodegradability. Padermshoke et al. [53] highlighted that such additives may introduce unintended ecological effects, underscoring the need for systematic evaluation of degradation promoters in marine environments.

Overall, effective microplastic mitigation requires a dual-driven approach combining scientific innovation and policy coordination. Scientific advances provide the technological pathways, whereas policy frameworks ensure large-scale implementation. Only through the integration of research, industry, and governance can sustainable and ecologically safe strategies for PP-MPs management be achieved.

## 5. Conclusions

PP-MPs are persistent pollutants in marine ecosystems due to their hydrophobic nature, chemical inertness, and structural stability. This review has examined the degradation mechanisms of PP-MPs, emphasizing the influence of polymer properties, environmental conditions, and microbial community dynamics. Despite the involvement of bacteria, fungi, and algae in oxidative modification, depolymerization, and mineralization, biodegradation rates in marine environments remain inherently slow. Although the photocatalytic process accelerates the breakdown, the complete mineralization of PP particles is estimated to require hundreds of hours or several months of continuous irradiation under current experimental conditions. In contrast, under natural environmental conditions without catalysts, this process would span decades to centuries. These processes are constrained by factors such as limited oxygen availability, low temperatures, and high salinity. While photochemical aging and mechanical abrasion enhance microbial colonization by introducing surface functional groups and increasing roughness, natural weathering alone is insufficient to counteract the scale of PP-MP accumulation.

To effectively mitigate PP-MP pollution, integrated strategies combining biological, material-based, and policy-driven approaches are essential. Advances in genetically engineered microbial consortia, multifunctional catalytic materials, and environmentally responsive degradation systems offer promising solutions for enhancing the degradation of PP-MPs while offering a potentially more efficient pathway for plastic mineralization. However, it is noted that the ecological safety of deploying engineered microbes requires rigorous assessment before practical application. Current research primarily focuses on the molecular mechanisms of degradation. Future studies should prioritize the development of scalable, practical applications for large-scale environmental remediation. Key research directions include the design of programmable degradable polymers, the integration of AI-based monitoring frameworks for real-time detection, and source control of PP-MPs.

In conclusion, a coordinated effort across scientific research, industrial innovation, and regulatory policy will be crucial to transitioning from passive remediation to proactive, ecosystem-based management of PP-MP pollution. This multi-disciplinary approach will support the development of sustainable solutions for PP-MP pollution control and contribute to the long-term health of marine ecosystems.

## Figures and Tables

**Table 1:** Main microorganisms on the surface of PP-MPs.

Main Microorganisms	Sampling Sites
*Flavobacteriales*, *Rhodobacterales*, *Chitinophagales*	Island of Elba in the Mediterranean Sea [14]
*Erythrobacte*	Mondego estuary [15]
*Flavobacteriaceae*, *Rhodobacteraceae*, *Rhodothermaceae*	marine aquaculture sites along the southeast coast of China [16]
*Pirellulaceae*, *Flavobacteriaceae*, *Rhodobacteraceae*,	Caribbean Sea [17]
*Psychrobacter*, *Pseudomonas*, *Flavobacterium*	Coastal area of Busan City [18]
*Burkholderiales*, *Enterobacterales*	Fal Estuary [19]
*Bacteroidia*, *Gammaproteobacteria*, *Alphaproteobacteria*	Mediterranean Sea [20]
*Bacteroidales*, *Verrucomicrobiales*, *Clostridiales*	Freshwater Lake of Hungary [21]
*Bacillus*, *Rhodococcus*	Peninsular Malaysia [22]
*Bacillus*, *Stenotrophomonas*, *Brucella*	coastal regions in the Indian state of Tamil Nadu [23]
*Alcanivorax*	the Coastal area of Kochi, Japan [24]
*Pseudomonas*, *Rhodococcus*	the Antarctic [25]

## Data Availability

No new data were created or analyzed in this study. Data sharing is not applicable to this article.
